# From Human Mesenchymal Stem Cells to Insulin-Producing Cells: Comparison between Bone Marrow- and Adipose Tissue-Derived Cells

**DOI:** 10.1155/2017/3854232

**Published:** 2017-05-11

**Authors:** Mahmoud M. Gabr, Mahmoud M. Zakaria, Ayman F. Refaie, Engy A. Abdel-Rahman, Asmaa M. Reda, Sameh S. Ali, Sherry M. Khater, Sylvia A. Ashamallah, Amani M. Ismail, Hossam El-Din A. Ismail, Nagwa El-Badri, Mohamed A. Ghoneim

**Affiliations:** ^1^Urology and Nephrology Center, Mansoura, Egypt; ^2^Zewail City of Science and Technology, Giza, Egypt; ^3^Department of Plastic Surgery, Mansoura University, Mansoura, Egypt

## Abstract

The aim of this study is to compare human bone marrow-derived mesenchymal stem cells (BM-MSCs) and adipose tissue-derived mesenchymal stem cells (AT-MSCs), for their differentiation potentials to form insulin-producing cells. BM-MSCs were obtained during elective orthotopic surgery and AT-MSCs from fatty aspirates during elective cosmetics procedures. Following their expansion, cells were characterized by phenotyping, trilineage differentiation ability, and basal gene expression of pluripotency genes and for their metabolic characteristics. Cells were differentiated according to a Trichostatin-A based protocol. The differentiated cells were evaluated by immunocytochemistry staining for insulin and c-peptide. In addition the expression of relevant pancreatic endocrine genes was determined. The release of insulin and c-peptide in response to a glucose challenge was also quantitated. There were some differences in basal gene expression and metabolic characteristics. After differentiation the proportion of the resulting insulin-producing cells (IPCs), was comparable among both cell sources. Again, there were no differences neither in the levels of gene expression nor in the amounts of insulin and c-peptide release as a function of glucose challenge. The properties, availability, and abundance of AT-MSCs render them well-suited for applications in regenerative medicine.* Conclusion*. BM-MSCs and AT-MSCs are comparable regarding their differential potential to form IPCs. The availability and properties of AT-MSCs render them well-suited for applications in regenerative medicine.

## 1. Introduction

According to a report by the WHO, 442 million people suffer from diabetes worldwide [1]. Type I DM is a scourge of young patients. It is the result of autoimmune destruction of pancreatic islets and accounts for 5–10% of diabetic patients. Lifelong insulin therapy is required for its control. An ideal alternative is transplantation of an intact pancreas or pancreatic islets. Scarcity of cadaveric organs and the need for immunosuppression, some of which are diabetogenic, are limiting factors.

Differentiation of stem cells from various sources to form insulin-producing cells (IPCs) provides a new and promising strategy to reconstitute pancreatic endocrine function. D'Amour and associates developed a multistep differentiation technique for the differentiation of human embryonic stem cells to form pancreatic progenitors [2]. A similar approach was also reported by Rezania et al. [3] and Pagliuca and coworkers [4]. Due to their teratogenicity and immunogenicity such cells have to be transplanted within an immunoisolation device. Differentiation of induced pluripotent stem cells (iPCs) can provide another useful source. Tateishi et al. were probably the first to report the possibility of generating insulin-secreting islet-like clusters from iPCs derived from human skin fibroblasts [5]. In a recent report, human iPCs were differentiated in vitro into pancreas-committed cells. At the end of in vitro differentiation approximately 5% of cells became insulin positive [6]. Mesenchymal stem cells (MSCs) can be derived from various sources. The bone marrow [7, 8], adipose tissue [9], umbilical cord [10], liver cells [11], and endometrium [12] are among several tissues that are rich in MSCs. Of these, the bone marrow and adipose tissues offer distinct advantages in view of their availability and abundance and the extent of their documentation.

In our laboratory, we have succeeded in the differentiation of human bone marrow-derived MSCs (BM-MSCs) to form insulin-producing cells (IPCs) [13, 14]. The aim of this study is to compare the adipose tissue-derived mesenchymal stem cells (AT-MSCs) and BM-MSCs regarding their potentials for differentiation to form IPCs.

## 2. Material and Methods

### 2.1. Recruitment of Mesenchymal Stem Cells (MSCs)

The required approvals for this study were obtained from the ethical committee of the University of Mansoura.

Bone marrow samples were obtained from two cases undergoing elective orthopedic surgical procedures at the Mansoura University Hospital. Liposuction aspirates were obtained from two healthy donors during elective cosmetic surgeries.

### 2.2. Isolation and Expansion of MSCs

Bone marrow samples were diluted 1 : 1 with low-glucose Dulbecco's Modified Eagle's Medium (DMEM, Sigma, St. Louis, Missouri, USA), layered on a top of a density gradient (Ficoll-Paque, 1.077 g/ml) (Pharmacia, Uppsala, Sweden) and centrifuged for 20 min at 600 ×g. The cells were collected from the DMEM/Ficoll interface, washed twice in phosphate buffer saline (PBS), and resuspended in 10 ml of low-glucose complete DMEM (supplemented with 10% fetal bovine serum (Hyclone, Logan, UT, USA), 100 U/mL penicillin, and 100 U/mL streptomycin (Sigma)). One milliliter of aspirates (BMA) yielded *≃*1.5 × 10^6^ nucleated cells. The collected cells were cultured in complete DMEM at a density of 5 × 10^5^ cells/mL (10 mL in 25 cm^2^ tissue culture flasks) and incubated at 37°C in 5% CO_2_ incubator.

The liposuction aspirates were digested by 0.075% collagenase type I (Sigma-Aldrich, St. Louis) for 30 minutes at 37°C with gentle stirring. The collagenase was inactivated with an equal volume of DMEM/10% fetal bovine serum (FBS) and centrifuged for 10 min at 300 ×g. The cellular pellet was resuspended in DMEM/10% FBS and filtered through a 100 *μ*m mesh filter to remove debris. The resuspended cells were plated at a density of 1 × 10^6^/cm^2^ into 75 cm^2^ culture flasks and incubated at 37°C in 5% CO_2_ incubator.

Three days thereafter, the nonadherent cells from both sources were discarded. The adherent cells were cultured to 80% confluence before passaging with trypsin. The cells were recultured in complete DMEM and replated at a ratio of 1 : 2 and cultured for another *≃*8 days to reach 80% confluence. The doubling time for both types of cells was determined and compared. This step was repeated for 3–5 passages. At this point, the cells were spindle-shaped and displayed a fibroblast-like appearance. The obtained sample from each donor was examined at least in triplicate.

### 2.3. Characterization of the Isolated MSCs

#### 2.3.1. Phenotyping

MSCs at passage 3 were trypsinized, centrifuged at 300 ×g for 8 min, and resuspended in PBS at a concentration of 1 × 10^6^ cells/mL. Aliquots of 100 *μ*L were incubated for 30 min in 20 *μ*L of antibodies against CD14/CD45 (FITC) or CD73/CD34 phycoerythrin (PE) or in 5 *μ*L of CD105 (PE) or CD90 (FITC) (Becton-Dickinson, USA), washed with 1 mL of stain buffer (BD-Pharmingen, USA), and resuspended in 500 *μ*L of stain buffer. The labeled cells were analyzed using an argon ion laser at a wave length of 488 nm (FACSCalibur, Becton-Dickinson, USA). A total of ten thousand events were obtained and analyzed using CellQuest software (Becton-Dickinson, USA). Control staining using the appropriate isotype-matched monoclonal antibodies was included.

### 2.4. Multilineage Differentiation Potential

MSCs from both sources were induced to differentiate into adipocytes, chondrocytes, and osteocytes using a previously described differentiation protocol [15]. Oil-Red-O was used to stain adipocytes; alcian blue was used to stain chondrocytes; and alizarin-red was used to stain osteocytes.

#### 2.4.1. Metabolic Analysis

This was carried out using the XF-24 extracellular flux analyzer (Agilent Technologies, Berlin, Germany) as previously described [16]. The expanded cells from both sources were seeded at a density of 40.000 cells/well in 24-well cell culture plates. The seeded cells were cultured in DMEM supplemented with 10% FBS and 1% penicillin and incubated at 37°C in a 5% CO_2_ incubator for 24 hours. The media were removed and replaced with the assay medium. For the mitochondrial stress assay (mito-stress assay), 300 *μ*L/well of the assay medium (10 mM glucose, 5 mM pyruvate, and 1 mM glutamine) is sued and left in a CO_2_ free preparation station for one hour. Thereafter, 50 *μ*L of the test reagents were sequentially injected into each well to obtain a final concentration of 4 *μ*M for each of the following: oligomycin (Sigma-Aldrich, Germany), carbonyl cyanide 3-chlorophenylhydrazone (CCCP, Sigma), and rotenone/antimycin mixture (Sigma). For the glycolytic-stress test, the culture medium was replaced by glucose-free DMEM and incubated at 37°C in 5% CO_2_ for 2 hours to induce substrate deprivation. Then 50 *μ*L of the test reagents was sequentially added to each well to give a final concentration of 5 mM glucose, 4 *μ*M oligomycin, and 40 mM 2-desoxy glucose (Sigma). Base line oxygen consumption rate (OCR) and the extracellular acidification rate (ECAR) were calculated based on the medium values obtained from 5 time points during the assay and were normalized by the viable cell count determined at the end of the test run.

#### 2.4.2. Differentiation of the MSCs into Endocrine Cells

Differentiation was performed according to a protocol previously reported by Tayaramma et al. [17]. Initially, the cells were cultured for 3 days in serum-free DMEM supplemented with Trichostatin-A (TSA) at a concentration of 55 nanomoles (Sigma-Aldrich Co., St. Louis. Missouri, USA). Then, the cells were cultured for an additional 7 days in high-glucose (25 millimoles) medium containing a 1 : 1 ratio of DMEM : DMEM/F12 (Sigma). This mixture was supplemented with 10% fetal bovine serum and 10 nanomoles' glucagon-like peptide-1 (GLP-1, Sigma).

#### 2.4.3. Immunofluorescence

Cell preparations were cultured on chamber slides (Nunc, Thermo Scientific, Rochester, NY). Then, the cells were fixed in 4% paraformaldehyde, permeabilized using chilled 100% methanol for 10 min, blocked with 5% normal goat serum for 60 min at RT, and incubated overnight in the primary antibodies at 4°C. Subsequently, the cells were washed with FBS and incubated in the secondary antibodies for 2 hours at RT. The nuclei were counterstained with DAPI. ImageJ software was used to determine the proportion of insulin-positive cells. To this end, ten fields were randomly selected for cell counting which was carried by two independent histopathologists. The results from all fields were calculated and expressed as the mean proportion of insulin-positive cells out of the total number of cells. In the above study, confocal images were captured using a Leica TCS SP8 microscope (Leica, Mannheim, Germany).

#### 2.4.4. Gene Expression by RT-qPCR

Total RNA was extracted from the undifferentiated MSCs as well as from cells at the end of in vitro differentiation using the RNeasy Plus Mini Kit (Qiagen GmbH, Hilden, Germany). Three micrograms of total RNA was converted into cDNA using the RT^2^ First-Strand Kit (Qiagen Science, Maryland, USA). Custom gene arrays were designed and supplied in 96-well plates (Qiagen Science). Before differentiation, gene expression for Nestin, PDX1, OCT4, Nanog, and SOX4 was evaluated, while, after differentiation, gene expression of the relevant pancreatic endocrine genes was determined. These included the following: insulin, glucagons, somatostatin, gult 2, glucokinase (GCK), RFX6, and neurod1. Amplifications were performed in each well using a 25-*μ*L reaction volume consisting of 12.5 *μ*L of 2x TaqMan Master Mix (RT^2^ SYBR Green qPCR, Qiagen Science), 1 *μ*L of cDNA template, and 11.5 *μ*L of nuclease-free water. The plate was inserted into a real-time thermal cycler (CFX96 Real-Time System, Bio-Rad, USA) that was programmed according to the manufacturer's instructions. The procedure was performed in triplicate for each sample. A mathematical model introduced by Pfaffl was used for the relative quantification of target genes [18]. In this study, gene expression of the undifferentiated MSCs was relative to GAPDH while that of the differentiated cells was relative to that of the undifferentiated ones.

#### 2.4.5. In Vitro Insulin and c-Peptide Release in Response to Increasing Glucose Concentrations

One million cells were initially incubated for 3 hours in glucose-free Krebs-Ringer bicarbonate buffer (KRB). This was followed by incubation for 1 hour in 3.0 mL of KRB containing 5.5, 12, or 25 mM glucose concentrations. The supernatant was collected at the end of each incubation period. The collected samples were frozen at −70°C until being assayed using an Elisa Kit with a minimum detection limit of 1.76 *μ*IU/ml (DRG Diagnostic, Germany).

#### 2.4.6. Statistical Analysis

Nonparametric data were evaluated by Friedman's test. Post hoc testing was preformed by Wilcoxon's Signed Ranking and *P* values were corrected by Bonferroni adjustments. A *P* value of <0.05 was considered significant.

## 3. Results

### 3.1. General Characteristics of MSCs

MSCs derived from the bone marrow or adipose tissue adhered to plastic and exhibited a spindle shape fibroblast-like morphology. The doubling time of AT_MSCs was *≃*48 hours while that of BM-MSCc was *≃*72 hours. The cells from both sources were strongly positive for the MSCs surface markers—CD73, CD90, and CD105—and were negative for the hematopoietic stem cell markers: CD14, CD34, and CD45 (Supplementary Data Table 1, in Supplementary Material available online at https://doi.org/10.1155/2017/3854232). In this study the results confirm that the isolated cells from these two tissues were indeed MSCs.

**Figure 1:**
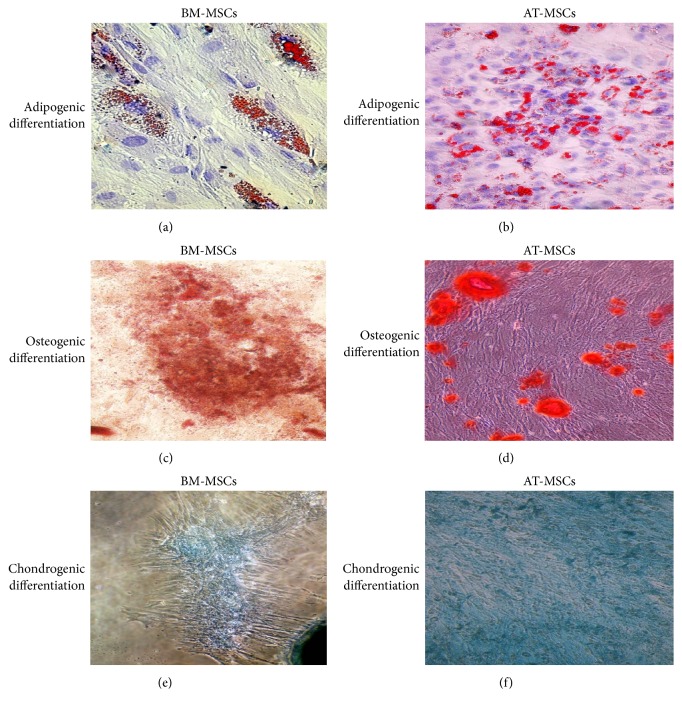
*Multilineage differentiation of BM-MSCs and AT-MSCs*. Adipogenesis was detected using Oil-Red-O staining (a, b), osteogenesis was detected using alizarin-red staining (c, d), and chondrogenesis was detected using Alcian blue (e, f).

The isolated cells from the bone marrow as well as those isolated from the fat tissue aspirates could be differentiated to form adipocytes, chondrocytes, and osteocytes when the appropriate growth factors were added (Figure 1).

### 3.2. The Metabolic Assay (Figure 2)

**Figure 2:**
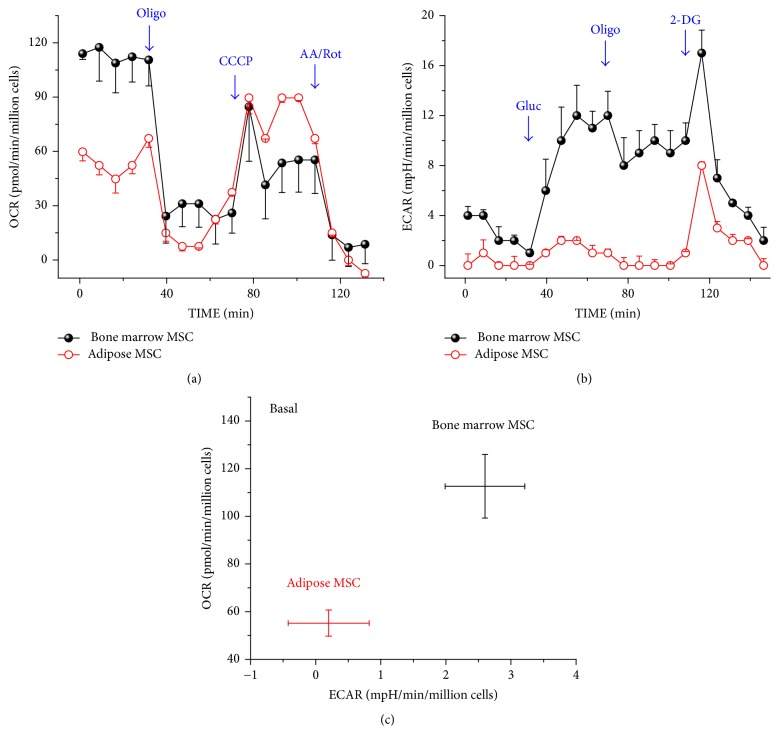
*The metabolic assay. The oxygen consumption rate (OCR) and the extracellular acidification rate (ECAR) were measured before and after adding the pharmacological agents to the respiring BM-MSCs (solid black circles) and AT-MSCs (open red circles)*: (a) the OCR profile. Oligomycin, an inhibitor of ATP synthesis, was added to block ATP synthesis by the mitochondria. CCCP, a protonophore, was added to drive the electron transport to its maximum rate. Finally, rotenone, a complex I inhibitor, and antimycin A, a complex III inhibitor, were added to block the electron transport chain. The nonmitochondrial oxygen consumption was left as a residual. (b) The ECAR profile. Initially glucose was added, followed by oligomycin and 2 deoxyglucose to inhibit glycolytic activities, thus leaving ECAR due to cellular nonglycolytic activities. (c) The basal metabolic profile. OCR (aerobic metabolism) and ECAR (glycolytic flux) were concurrently measured and compared among both types of cells.

In this study, the endogenous oxygen consumption rate (OCR) and the extracellular acidification rate (ECAR) were determined for both types of cells under basal, stimulated, or inhibited states. Both basal mitochondrial OCR and glycolytic activity by BM-MSCs were higher relative to that of AT-MSCs (Figures 2(a) and 2(b)). However AT-MSCs exhibited greater reserve mitochondrial capacity calculated by subtraction of the basal OCR from the OCR after the addition of CCCP (Figure 2(a)). Collectively, the basal metabolic activities including oxidative phosphorylation and glycolysis were greater among BM-MSCs (Figure 2(c)). However, AT-MSCs appear to possess and depend on mitochondrial bioenergetics especially under stimulated conditions.

### 3.3. Immunofluorescence

**Figure 3:**
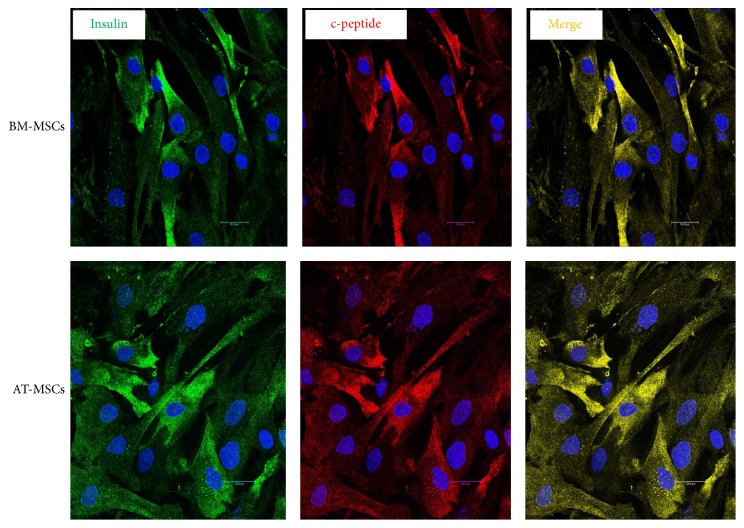
*Immunofluorescence of BM-MSCs and AT-MSCs following in vitro differentiation*. Both types of cells were positive for insulin (gene) and c-peptide (red). Insulin and c-peptide were coexpressed in the same cells (yellow).

By immunofluorescence, the proportion of insulin-positive cells at the end of differentiation ranged between 1.0 and 5% for BM-MSCs and between 1.0 and 3.4% for AT-MSCs. By flow cytometry incidence ranged between 0.4 and 3.4% for BM-MSCs and 0.8 and 4.1% for AT-MSCs (Figure 3). Differences did not have any statistical difference by either method (Supplementary Data, Table 2).

### 3.4. Relative Gene Expression

**Figure 4:**
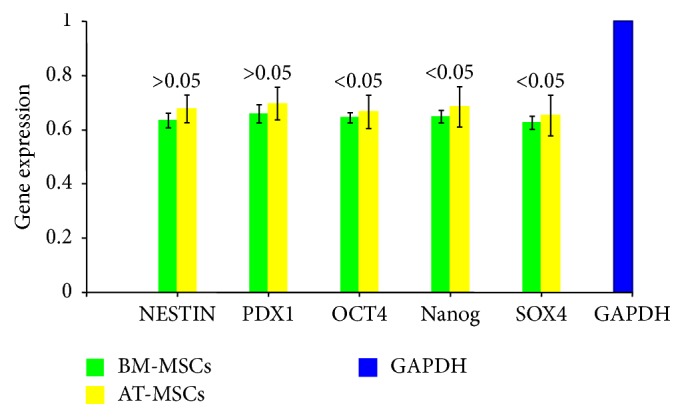
*Relative expression of relevant genes by BM-MSCs and AT-MSCs before differentiation*. The expression of nestin and PDX1 was comparable among the MB-MSCs and AT-MSCs (*P* > 0.05). The expressions of OCT4, Nanog, and SOX4 were significantly higher among AT-MSCs (*P* < 0.05).

We have studied the relative gene expression of BM-MSCs and AT-MSCs before their differentiation. Emphasis was placed on the evaluation of some genes which suggest a potential for possible differentiation towards pancreatic endocrine lineage (Figure 4). The expression levels of PDX1 and nestin were comparable among the two cell types. On the other hand, expression of OCT4, Nanog, and SOX4 was higher in the AT-MSCs (Supplementary Data, Table 3).

**Figure 5:**
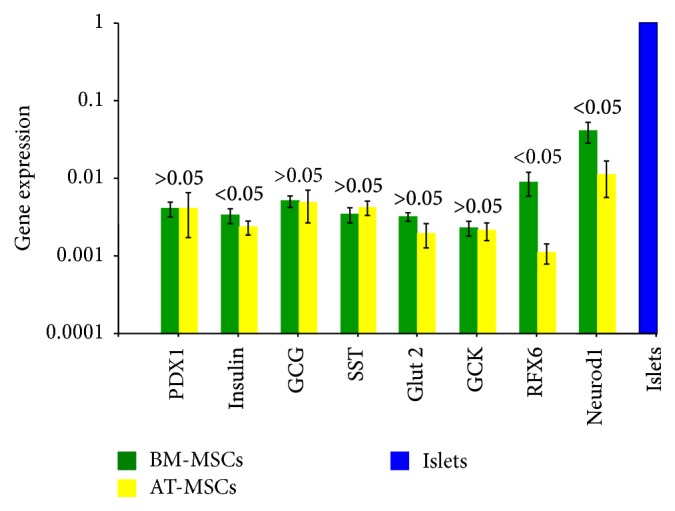
*Relative expression of relevant genes by BM-MSCs and AT-MSCs after differentiation*. The expression of PDX1, glucagons, somatostatin, GLUT 2, and GCK was similar among the cells of both tissues (*P* > 0.05). After differentiation, the expression of insulin, RFX6, and Neurod1 was higher among BM-MSCs (*P* < 0.05).

After differentiation, the expression of PDX1, glucagon, somatostatin, GULT 2, and GCK was comparable in both BM-MSCs and AT-MSCs without a significant statistical difference. On the other hand expression of insulin, RFX6, and Neurod1 was higher among BM-MSCs (Figure 5). (Supplementary Data, Table 4).

### 3.5. In Vitro Human Insulin and c-Peptide Release in Sequence of Glucose Challenges

**Figure 6:**
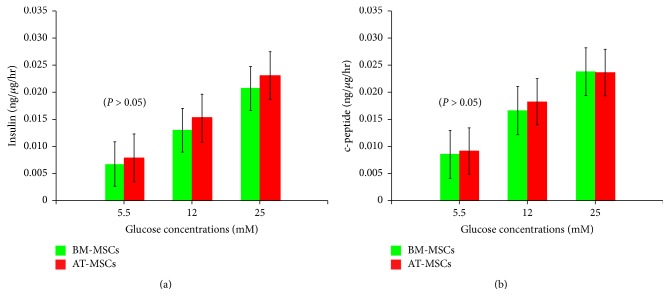
*In vitro human insulin and c-peptide release in response to glucose challenge*. The differentiated BM-MSCs and AT-MSCs released increasing amounts of insulin (a) and c-peptide (b) in response to increasing glucose concentrations (*P* < 0.05). The amounts of insulin and c-peptide at different concentrations of glucose were comparable among both types of cells (*P* > 0.05).

Upon differentiation of BM-MSCs and AT-MSCs into IPCs, the differentiated cells from both tissues released increasing amounts of insulin and c-peptide in response to increasing glucose concentrations (Figures 6(a) and 6(b)). However there was no statistical difference between the amount of insulin and c-peptide released by cells obtained from bone marrow or adipose tissue at any given concentration (Supplementary Tables 5(a)–5(D)).

## 4. Discussion

The ability to purify, culture, and differentiate stem cells from nonembryonic origin can provide an important cell sources for regenerative medicine. The term mesenchymal stem cells was coined by Caplan to refer to plastic-adherent cell populations isolated from a variety of postnatal and adult tissues [19]. Nevertheless, more recent studies concluded that convincing data to support the stemness of these unfractionated plastic adhering cells are lacking [20]. The term mesenchymal stromal cells was suggested allowing the abbreviation “MSCs” to be maintained. Several independent studies have demonstrated that MSCs can differentiate not only into mesenchymal but also into ectodermal and endodermal lineages [21]. Based on these findings, the term multipotent mesenchymal stromal cells appears to be the most accurate descriptor [22]. The term mesenchymal is kept to imply their origin but not the differentiation potentials. These cells have a high capacity to replicate, are easy to cultivate, expand, and can maintain their multilineage potential following prolonged culture conditions [15]. Furthermore, they are nonteratogenic and their utilization is free of any ethical considerations.

MSCs derived from various sources were coaxed to differentiate into IPCs [7–11, 23]. Of these, bone marrow and adipose tissue offer distinct advantages in view of their availability and the extent of their documentation in literature.

In this study, cells obtained from bone marrow or adipose tissues were initially characterized relative to their morphology, phenotypic characteristics, proliferation rate, and their multilineage differentiation capability. After passage 3, both types of cells became homogenous and showed a fibroblast-like morphology with abundant cytoplasm and large nuclei. No significant differences in phenotypic characteristics were observed among both types of cells. This is in agreement with several other studies [24, 25]. However, we have observed that the proliferation rate among AT-MSCs was higher than that of BM-MSCs. This is in agreement with other published data [21, 26–28]. The multilineage differentiation potential of both types of cells was confirmed. Cells from both sources could be differentiated to form adipocytes, chondrocytes, and osteocytes when the appropriate growth factors were added. In a study by Li et al., it was reported that BM-MSCs exhibit superior capacity for osteogenic and chondrogenic differentiation but a similar capacity for adipogenic differentiation when compared with AT-MSCs [25]. Collectively, the abovementioned findings confirm that in this study cells from both sources are indeed MSCs and satisfy the requirements of the International Society for Cellular Therapy [22].

We have studied the relative gene expression of BM-MSCs and AT-MSCs before their differentiation, and emphasis was on the evaluation of some genes which suggest a potential for possible differentiation towards pancreatic endocrine lineage. The expression of PDX1, an important gene for ß-cell development, and Nestin, an endocrine precursor gene, was comparable among the two cell types. On the other hand, expression of the pluripotency genes—Oct4, Nanog and SOX4—was significantly higher in the AT-MSCs. These findings are in agreement with a study by Trivanović and associates [29]. They reported that expression of Nanog, Oct-4A, and SOX-2 is more pronounced in AT-MSCs. This may reflect that at least a subpopulation of AT-MSCs has trilineage potentials. After differentiation, all the relevant pancreatic endocrine genes were expressed by both types of cells. However, BM-MSCs expressed higher levels of insulin, RFX6, and Neurod1.

To the best of our knowledge, studies of possible differences in the metabolic characteristics between BM-MSCs and AT-MSCs are lacking. It has been increasingly clear that the switch of energy supply from glycolysis to aerobic metabolism is essential for successful differentiation of MSCs [30]. Chen et al. reported that the metabolic signature of stem cells correlates with the self-renewal status (high glycolytic flux) and the differential potential (mitochondrial function) [31]. In our own metabolic study, evidence was provided that the glycolytic activity of BM-MSCs was higher than that of AT-MSCs. This would favor a higher proliferation rate and consequently a shorter doubling time by BM-MSCs. Nevertheless, we had observed that AT-MSCs had a shorter doubling time. This may be explained by the findings of El-Badawy and associates [32]. These authors studied the telomerase activity in cell lysates from both types of cells. To this end, they used a PCR-based telomerase activity detection method and reported that there was a significantly higher telomerase activity among AT-MSCs. In addition, our metabolic studies revealed that AT-MSCs exhibited a greater mitochondrial capacity and relied on mitochondrial bioenergetics which are required during the differentiation phase. This advantage can be also supported in view of the higher expression of the pluripotency genes by AT-MSCs.

Based on our previous comparative study, a Trichostatin-A based protocol was employed for the in vitro differentiation of MSCs into insulin-producing cells (IPCs). This method was chosen in view of its simplicity and the short duration required for its completion [14]. The yield of IPCs was modest and comparable among both types of cells. This is in agreement with other studies which indicated that the proportion of IPCs at the end of in vitro differentiation was small irrespective of the method employed. The presence of insulin and c-peptide in the cytoplasm of the same cells confirms the intrinsic synthesis of insulin. All the important pancreatic endocrine genes were expressed. Again, no differences were observed among bone marrow-derived and adipose tissue-derived stem cells. There was a stepwise increase in insulin as well as c-peptide release by both types of cells as a function of increasing glucose concentrations. This indicates that the differentiated cells were glucose-sensitive and insulin-responsive. Several investigators had reported that although the proportion of IPCs generated in vitro from MSCs was meager they could induce euglycemia when transplanted in diabetic nude mice [33, 34]. In a previous study, we have provided evidence that these cells undergo further differentiation in vivo to reach a valve of *≃*20% four weeks after transplantation [35]. It seems that the in vivo environment serves to induce the cells towards a pancreatic endocrine lineage. Further maturation occurs after their transplantation under the influence of factors present in the in vivo milieu.

While BM-MSCs and AT-MSCs share many biological characteristics, there were some differences in their proliferation rate, basal gene expression, and metabolic characteristics. Strioga et al. reported some additional differences in their immunophenotype and immunomodulatory activity [36]. In a study by Marappagounder and associates, it was reported that BM-MSCs are more promising in transdifferentiation into pancreatic islet-like cluster compared to AT-MSCs [37]. However, their conclusion was not supported by the provided results. Gene expression of their differentiated islet-like cluster did not reveal a statistical difference between the two cell sources. Again, the insulin release as a function of increasing glucose challenge was also comparable.

## 5. Conclusion

In our study, it was clear that BM-MSCs and AT-MSCs are comparable in terms of the proportion of generated insulin-producing cells and relative gene expression of pancreatic endocrine genes and insulin and c-peptide release as a result of a glucose challenges. The clinical applicability of BM-MSCs may be limited due to the invasive procedure required for sample collection. On the other hand, liposuction aspirates are widely available and should not be wasted. Furthermore, one gram of adipose tissue yields *≃*5000 stem cells, whereas the yield from bone marrow is only 100–1000 cells/ml [38]. Collectively, the properties of AT-MSCs render them well-suited for applications in regenerative medicine and can provide a viable alternative for BM-MSCs.

## Supplementary Material


**Table (1):** The cells from both sources were strongly positive for the MSCs surface markers: CD73, CD90 and CD105 and were negative for the hematopoietic stem cell markers; CD14, CD 34 and CD 45.
**Table (2):** By immunofluorescence, the proportion of insulin-positive cells at the end of differentiation ranged between1.0–5% for BM-MSCs and between 1.0–3.4% for AT-MSCs.
**Table (3):** The expression levels of PDX1 and nestin, were comparable among the two cell types. On the other hand, expression of OCT4, Nanog and SOX4 was higher in the AT-MSCs.
**Table (4):** After differentiation, the expression of PDX1, glucagon, somatostatin, GULT2 and GCK was comparable in both BM-MSCs and AT-MSCs without a significant statistical difference. On the other hand expression of insulin, RFX 6 and Neurod1 was higher among BM-MSCs.
**Table (5-A):** There was a stepwise increase of insulin release from differentiated BM-MSCs as a response of increasing glucose constrictions.
**Table (5-B):** There was a stepwise increase of insulin release from differentiated AT-MSCs as a response of increasing glucose constrictions.
**Table (5-C):** There was a stepwise increase c-peptide release from differentiated BM-MSCs as a response of increasing glucose constrictions.
**Table (5-D):** There was a stepwise increase of c-peptide release from differentiated At-MSCs as a response of increasing glucose constrictions.
