# Supplementary material for: From Human Mesenchymal Stem Cells to Insulin-Producing Cells: Comparison between Bone Marrow- and Adipose Tissue-Derived Cells
Source: Biomed Res Int. 2017 May 11;2017:3854232. doi: 10.1155/2017/3854232 (PMC5444016; doi:10.1155/2017/3854232)
Supplement: Supplementary file 1 — Table (1): The cells from both sources were strongly positive for the MSCs surface markers: CD73, CD90 and CD105 and were negative for the hematopoietic stem cell markers; CD14, CD 34 and CD 45. Table (2): By immunofluorescence, the proportion of insulin-positive cells at the end of differentiation ranged between1.0–5% for BM-MSCs and between 1.0–3.4% for AT-MSCs. Table (3): The expression levels of PDX1 and nestin, were comparable among the two cell types. On the other hand, expression of OCT4, Nanog and SOX4 was higher in the AT-MSCs. Table (4): After differentiation, the expression of PDX1, glucagon, somatostatin, GULT2 and GCK was comparable in both BM-MSCs and AT-MSCs without a significant statistical difference. On the other hand expression of insulin, RFX 6 and Neurod1 was higher among BM-MSCs. Table (5-A): There was a stepwise increase of insulin release from differentiated BM-MSCs as a response of increasing glucose constrictions. Table (5-B): There was a stepwise increase of insulin release from differentiated AT-MSCs as a response of increasing glucose constrictions. Table (5-C): There was a stepwise increase c-peptide release from differentiated BM-MSCs as a response of increasing glucose constrictions. Table (5-D): There was a stepwise increase of c-peptide release from differentiated At-MSCs as a response of increasing glucose constrictions. [file 3854232.f1.doc]

**Supplementary data (table 1)**

**Phenotype Characteristics**

|  | **Bone marrow MSCs (BM-MSCs)** | | | | | |  | **Adipose Tissue MSCs (AT-MSCs)** | | | | | |
| --- | --- | --- | --- | --- | --- | --- | --- | --- | --- | --- | --- | --- | --- |
|  | **CD-73**  **(%)** | **CD-90**  **(%)** | **CD-105**  **(%)** | **CD-14**  **(%)** | **CD-34**  **(%)** | **CD-45**  **(%)** |  | **CD-73**  **(%)** | **CD-90**  **(%)** | **CD-105**  **(%)** | **CD-14**  **(%)** | **CD-34**  **(%)** | **CD-45**  **(%)** |
| **Donor 1** | 99.95  97.04  99.03 | 98.05  94.04  83 | 99.9  91.7  96.4 | 0.07  4.07  0.07 | 0.08  0.01  0.05 | 0.05  3.04  1.04 | **Donor 3** | 99.9  98  98.7 | 98.5  91  99.1 | 97.6  97  98.9 | 0.03  0.05  0.58 | 3  0.05  0.82 | 9.09  0.05  0.19 |
| **Donor 2** | 98.05  90.03  97.08 | 97.8  90  95.5 | 96.4  94.6  98.6 | 0.06  0.07  0.07 | 0.0  0.1  0.9 | 0.00  0.05  0.01 | **r**  **Donor 4**  **4** | 93.9  95.1  90.8 | 99.2  81.6  29.9 | 88.5  97.7  91.7 | 0.02  0.05  0.59 | 0.76  0.06  0.07 | 0.16  0.05  0.04 |
| **Mean**  **St. Error** | **97.2**  **1.4** | **93.3**  **2.4** | **96.3**  **2.9** | **1.2**  **0.6** | **0.2**  **0.1** | **0.8**  **0.5** |  | **96.1**  **1.4** | **93.2**  **2.8** | **95.2**  **1.7** | **0.04**  **0.06** | **0.9**  **0.3** | **1.73**  **3.61** |

**Supplementary data (table 2)**

**Percentage of Insulin positive cells generated**

**at end of differentiation (immunofluorescence)**

| **Mean + S.D.** | **BM-MSCs** | **AT-MSCs** |
| --- | --- | --- |
|  | **3.4**  **5.0**  **1.0**  **1.47**  **2.8**  **3.6**  **1.8**  **2.4**  **1.9** | **2.7**  **2.5**  **1.0**  **3.2**  **3.4**  **2.9**  **1.8**  **2.1**  **1.8** |
|  | **2.60 + 1.25** | **2.38 + 0.77** |

***P = 0.83***

**Supplementary data (Table 3)**

Relative expression of relevant genes before differentiation (BM-MSCs).

|  | **Donor 1** | | | | | | **Donor 2** | | | | | | ***Mean ± S.E.*** |
| --- | --- | --- | --- | --- | --- | --- | --- | --- | --- | --- | --- | --- | --- |
| **nestin** | 0.6193 | 0.6325 | 0.6389 | 0.6396 | 0.6293 | 0.6426 | 0.6876 | 0.6894 | 0.6988 | 0.6614 | 0.6614 | 0.6595 | ***0.6550±.0075*** |
| **pdx1** | 0.6376 | 0.6424 | 0.6446 | 0.6691 | 0.6743 | 0.6805 | 0.6664 | 0.6744 | 0.7222 | 0.7222 | 0.7236 | 0.6693 | ***0.6816±.0096*** |
| **oct4** | 0.6331 | 0.6297 | 0.6265 | 0.6448 | 0.6623 | 0.6563 | 0.6456 | 0.6479 | 0.6559 | 0.6901 | 0.6901 | 0.6621 | ***0.6548±.0057*** |
| **nanog** | 0.6329 | 0.6381 | 0.6320 | 0.6697 | 0.6584 | 0.6566 | 0.6817 | 0.6637 | 0.6786 | 0.6882 | 0.6882 | 0.7027 | ***0.6659±.0067*** |
| **sox4** | 0.6085 | 0.6250 | 0.6221 | 0.6101 | 0.6308 | 0.6576 | 0.6243 | 0.6393 | 0.6347 | 0.6656 | 0.6656 | 0.6808 | ***0.6387±.0068*** |
| **GAPDH** | 1 | 1 | 1 | 1 | 1 | 1 | 1 | 1 | 1 | 1 | 1 | 1 | ***1*** |

**Relative expression of relevant genes before differentiation (AT**-MSCs)

|  | **Donor 3** | | | | | | **Donor 4** | | | | | | ***Mean ± S.E.*** |
| --- | --- | --- | --- | --- | --- | --- | --- | --- | --- | --- | --- | --- | --- |
| **nestin** | 0.6876 | 0.6894 | 0.6988 | 0.5925 | 0.6173 | 0.6249 | 0.7304 | 0.7385 | 0.7220 | 0.7033 | 0.7156 | 0.7359 | ***0.6880±.0143*** |
| **pdx1** | 0.6664 | 0.6695 | 0.6744 | 0.6476 | 0.6495 | 0.6557 | 0.7280 | 0.7392 | 0.7360 | 0.7932 | 0.7907 | 0.8032 | ***0.7128±.01723*** |
| **oct4** | 0.6456 | 0.6479 | 0.6559 | 0.6196 | 0.6334 | 0.6339 | 0.7688 | 0.7714 | 0.7609 | 0.7188 | 0.7136 | 0.7746 | ***0.6954±.01790*** |
| **nanog** | 0.6817 | 0.6637 | 0.6786 | 0.6414 | 0.6396 | 0.6481 | 0.8081 | 0.8025 | 0.8046 | 0.7811 | 0.7981 | 0.8122 | ***0.7280±.02185*** |
| **sox4** | 0.6243 | 0.6393 | 0.6347 | 0.6017 | 0.6115 | 0.6165 | 0.7421 | 0.7465 | 0.7303 | 0.7793 | 0.7713 | 0.7882 | ***0.6905±.02150*** |
| **GAPDH** | 1 | 1 | 1 | 1 | 1 | 1 | 1 | 1 | 1 | 1 | 1 | 1 | ***1*** |

**Supplementary data (Table 4)**

**Relative expression of relevant genes before differentiation (AT-MSCs)**

|  | **Donor 1** | | | | | | **Donor 2** | | | | | | ***Mean ± S.E.*** |
| --- | --- | --- | --- | --- | --- | --- | --- | --- | --- | --- | --- | --- | --- |
| **PDX1** | 0.0027 | 0.0024 | 0.0023 | 0.0016 | 0.0074 | 0.0119 | 0.0046 | 0.0058 | 0.0011 | 0.0032 | 0.0011 | 0.0051 | **0.0041±.0009** |
| **INS** | 0.0086 | 0.0068 | 0.0027 | 0.0033 | 0.0057 | 0.0052 | 0.0009 | 0.0013 | 0.0019 | 0.0021 | 0.0008 | 0.0007 | **0.0033±.0008** |
| **GCG** | 0.0105 | 0.0091 | 0.0027 | 0.0027 | 0.0073 | 0.0096 | 0.0035 | 0.0038 | 0.0020 | 0.0054 | 0.0022 | 0.0018 | **0.0051±.0009** |
| **SST** | 0.0025 | 0.0023 | 0.0013 | 0.0044 | 0.0080 | 0.0089 | 0.0023 | 0.0043 | 0.0015 | 0.0016 | 0.0017 | 0.0022 | **0.0034±.0007** |
| **Glut2** | 0.0037 | 0.0038 | 0.0030 | 0.0041 | 0.0046 | 0.0049 | 0.0041 | 0.0048 | 0.0015 | 0.0022 | 0.0014 | 0.0006 | **0.0032±.0004** |
| **GCK** | 0.0051 | 0.0049 | 0.0007 | 0.0006 | 0.0016 | 0.0041 | 0.0021 | 0.0033 | 0.0018 | 0.0025 | 0.0002 | 0.0002 | **0.0022±.0005** |
| **RFx6** | 0.0075 | 0.0100 | 0.0020 | 0.0029 | 0.0102 | 0.0395 | 0.0043 | 0.0106 | 0.0046 | 0.0109 | 0.0012 | 0.0012 | **0.0088±.0030** |
| **Neurod1** | 0.1190 | 0.1190 | 0.0170 | 0.0490 | 0.0344 | 0.0248 | 0.0629 | 0.0187 | 0.0118 | 0.0187 | 0.0025 | 0.0060 | **0.0403±.0117** |

**Relative expression of relevant genes after differentiation (BM-MSCs )**

|  | **Donor 3** | | | | | | **Donor 4** | | | | | | **Mean ± S.E.** |
| --- | --- | --- | --- | --- | --- | --- | --- | --- | --- | --- | --- | --- | --- |
| **PDX1** | 0.0008 | 0.0004 | 0.0009 | 0.0013 | 0.0007 | 0.0007 | 0.0033 | 0.0030 | 0.0023 | 0.0299 | 0.0019 | 0.0038 | **0.0041±.0024** |
| **INS** | 0.0040 | 0.0020 | 0.0014 | 0.0012 | 0.0014 | 0.0040 | 0.0017 | 0.0011 | 0.0025 | 0.0012 | 0.0019 | 0.0057 | **0.0023±.0004** |
| **GCG** | 0.0007 | 0.0013 | 0.0032 | 0.0033 | 0.0065 | 0.0013 | 0.0011 | 0.0014 | 0.0016 | 0.0284 | 0.0076 | 0.0018 | **0.0048±.0022** |
| **SST** | 0.0030 | 0.0019 | 0.0066 | 0.0072 | 0.0030 | 0.0092 | 0.0013 | 0.0014 | 0.0022 | 0.0042 | 0.0008 | 0.0095 | **0.0042±.0009** |
| **Glut2** | 0.0073 | 0.0007 | 0.0028 | 0.0034 | 0.0004 | 0.0010 | 0.0049 | 0.0004 | 0.0007 | 0.0003 | 0.0003 | 0.0002 | **0.0019±.0007** |
| **GCK** | 0.0023 | 0.0002 | 0.0055 | 0.0063 | 0.0016 | 0.0027 | 0.0011 | 0.0006 | 0.0016 | 0.0012 | 0.0013 | 0.0012 | **0.0021±.0005** |
| **RFx6** | 0.0029 | 0.0002 | 0.0034 | 0.0010 | 0.0001 | 0.0003 | 0.0023 | 0.0006 | 0.0014 | 0.0004 | 0.0005 | 0.0002 | **0.0011±.0003** |
| **Neurod1** | 0.0698 | 0.0086 | 0.0054 | 0.0083 | 0.0024 | 0.0041 | 0.0067 | 0.0099 | 0.0107 | 0.0019 | 0.0016 | 0.0022 | **0.0109±.0054** |

**Supplementary data (table 5-A)**

**Human insulin release from differentiated BM-MSCs**

| **Glucose Conc. (nM) / Insulin Conc. (ng/µg/hr)** | **5.5** | **12** | **25** |
| --- | --- | --- | --- |
|  | 0.007 | 0.015 | 0.025 |
|  | 0.007 | 0.012 | 0.023 |
|  | 0.006 | 0.014 | 0.02 |
|  | 0.009 | 0.016 | 0.024 |
|  | 0.006 | 0.011 | 0.02 |
|  | 0.008 | 0.015 | 0.026 |
|  | 0.007 | 0.014 | 0.02 |
|  | 0.006 | 0.013 | 0.02 |
|  | 0.008 | 0.014 | 0.022 |
|  | 0.006 | 0.012 | 0.016 |
|  | 0.004 | 0.01 | 0.013 |
|  | 0.006 | 0.009 | 0.019 |
| **Mean + S.E.** | **0.0067 + 0.0004** | **0.013+ 0.0006** | **0.021 + 0.001** |

**Supplementary data (table 5-B)**

**Human insulin release from differentiated AT-MSCs**

| **Glucose Conc. (nM) / Insulin Conc. (ng/µg/hr)** | **5.5** | **12** | **25** |
| --- | --- | --- | --- |
|  | 0.01 | 0.025 | 0.04 |
|  | 0.008 | 0.014 | 0.02 |
|  | 0.007 | 0.016 | 0.022 |
|  | 0.008 | 0.015 | 0.024 |
|  | 0.007 | 0.013 | 0.022 |
|  | 0.009 | 0.017 | 0.026 |
|  | 0.008 | 0.015 | 0.02 |
|  | 0.007 | 0.012 | 0.019 |
|  | 0.007 | 0.013 | 0.02 |
|  | 0.009 | 0.012 | 0.02 |
|  | 0.006 | 0.014 | 0.021 |
|  | 0.007 | 0.016 | 0.022 |
| **Mean + S.E.** | **0.0078 + 0.0003** | **0.015+ 0.001** | **0.023 + 0.00016** |

**Supplementary data (table 5-C)**

**Human C-peptide release from differentiated BM-MSCs**

| **Glucose Conc. (nM) / C-peptide Conc. (ng/µg/hr)** | **5.5** | **12** | **25** |
| --- | --- | --- | --- |
|  | 0.008 | 0.017 | 0.03 |
|  | 0.009 | 0.018 | 0.028 |
|  | 0.008 | 0.016 | 0.024 |
|  | 0.008 | 0.017 | 0.022 |
|  | 0.01 | 0.019 | 0.022 |
|  | 0.01 | 0.02 | 0.028 |
|  | 0.008 | 0.017 | 0.024 |
|  | 0.008 | 0.015 | 0.021 |
|  | 0.009 | 0.016 | 0.023 |
|  | 0.009 | 0.015 | 0.021 |
|  | 0.007 | 0.012 | 0.02 |
|  | 0.008 | 0.016 | 0.02 |
| **Mean + S.E.** | **0.0085 + 0.0003** | **0.0165+ 0.0005** | **0.024 + 0.0009** |

**Supplementary data (table 5-D)**

**Human C-peptide release from differentiated AT-MSCs**

| **Glucose Conc. (nM) / C-peptide Conc. (ng/µg/hr)** | **5.5** | **12** | **25** |
| --- | --- | --- | --- |
|  | 0.009 | 0.021 | 0.03 |
|  | 0.009 | 0.018 | 0.023 |
|  | 0.008 | 0.019 | 0.026 |
|  | 0.01 | 0.019 | 0.024 |
|  | 0.009 | 0.018 | 0.023 |
|  | 0.01 | 0.022 | 0.026 |
|  | 0.009 | 0.017 | 0.023 |
|  | 0.009 | 0.016 | 0.02 |
|  | 0.008 | 0.018 | 0.022 |
|  | 0.01 | 0.015 | 0.02 |
|  | 0.008 | 0.017 | 0.023 |
|  | 0.009 | 0.018 | 0.023 |
| **Mean + S.E.** | **0.009 + 0.0002** | **0.018+ 0.0006** | **0.024 + 0.0008** |
